# Limited impact on glucose homeostasis of leptin receptor deletion from insulin- or proglucagon-expressing cells

**DOI:** 10.1016/j.molmet.2015.06.007

**Published:** 2015-06-25

**Authors:** Helen Soedling, David J. Hodson, Alice E. Adrianssens, Fiona M. Gribble, Frank Reimann, Stefan Trapp, Guy A. Rutter

**Affiliations:** 1 Section of Cell Biology and Functional Genomics, Division of Diabetes, Endocrinology and Metabolism, Department of Medicine, Imperial College London, du Cane Road, London W12 0NN, UK; 2 University of Cambridge Metabolic Research Laboratories, Cambridge, UK; 3 Centre for Cardiovascular and Metabolic Neuroscience, Department of Neuroscience, Physiology & Pharmacology, University College London, London, UK

**Keywords:** Leptin, Insulin, Glucagon, GLP-1, Diabetes, β cell, α cell, L-cell, AUC, area under the curve, [Ca^2+^]_i_, intracellular free Ca^2+^ concentration, K_ATP_, ATP-sensitive K^+^ channel, GTT, ITT, glucose and insulin tolerance test, respectively, IP, intraperitoneal, NTS, nucleus tractus solitarius

## Abstract

**Aims/hypothesis:**

The adipose tissue-derived hormone leptin plays an important role in the maintenance of body weight and glucose homeostasis. Leptin mediates its effects by interaction with leptin receptors (LepRb), which are highly expressed in the hypothalamus and other brain centres, and at lower levels in the periphery. Previous studies have used relatively promiscuous or inefficient *Cre* deleter strains, respectively, to explore the roles of LepR in pancreatic β and α cells. Here, we use two newly-developed *Cre* lines to explore the role of leptin signalling in insulin and proglucagon-expressing cells.

**Methods:**

Leptin receptor expression was measured in isolated mouse islets and highly-purified islet cells by RNASeq and quantitative RT-PCR. Mice lacking leptin signalling in pancreatic β, or in α and other proglucagon-expressing cells, were generated using Ins1*Cre-* or iGlu*Cre*-mediated recombination respectively of *flox*'d leptin receptor alleles. *In vivo* glucose homeostasis, changes in body weight, pancreatic histology and hormone secretion from isolated islets were assessed using standard techniques.

**Results:**

Leptin receptor mRNA levels were at or below the level of detection in wild-type adult mouse isolated islets and purified cells, and leptin signalling to Stat3 phosphorylation was undetectable. Whereas male mice further deleted for leptin receptors in β cells exhibited no abnormalities in glucose tolerance up to 16 weeks of age, females transiently displayed improved glucose tolerance at 8 weeks (11.2  ±  3.2% decrease in area under curve; p < 0.05), and improved (39.0  ±  13.0%, P < 0.05) glucose-stimulated insulin secretion *in vitro.* No differences were seen between genotypes in body weight, fasting glucose or β/α cell ratio. Deletion of LepR from α-cells, a minority of β cells, and a subset of proglucagon-expressing cells in the brain, exerted no effects on body weight, glucose or insulin tolerance, nor on pancreatic hormone secretion assessed *in vivo* and *in vitro.*

**Conclusions/interpretation:**

The use here of a highly selective *Cre* recombinase indicates that leptin signalling plays a relatively minor, age- and sex-dependent role in the control of β cell function in the mouse. No *in vivo* role for leptin receptors on α cells, nor in other proglucagon-expressing cells, was detected in this study.

## Highlights

• β cell leptin receptors play a minor role in glucose homeostasis.
• α cell leptin receptors are not involved in responses to hypoglycaemia.
• Body weight maintenance does not require leptin action on GLP-1-secreting cells.

## 1 Introduction

Type 2 Diabetes mellitus (T2D) currently affects approximately 380 million individuals worldwide [1] and is characterized by elevated blood glucose levels. The treatment of T2D complications, which include cardiovascular disease, retinopathy and peripheral nerve damage, typically consumes 10–20% of the health care budgets of westernized societies [2]. Whilst insulin, secreted from pancreatic islet β cells, acts to lower blood glucose levels, glucagon, secreted by pancreatic α cells, increases glycaemia. Defects in the release or actions of either hormone can thus contribute to the disease [3].

Obesity, which affects ∼1 in 4 adults in the UK (www.hscic.gov.uk/catalogue/PUB10364), is an important risk factor for T2D and promotes both insulin resistance and β cell failure [4]. The adipose tissue-derived hormone leptin is an important satiety factor which acts on the feeding centres in the brain to suppress appetite [5]. Human mutations in either the leptin (obese, or *ob*) [6,7] or the leptin receptor (*LepR*) [8] gene lead to severe obesity from an early age. Demonstrating a conserved role for the hormone across mammalian species, mice carrying mutations in the homologous genes (*ob/ob* [9] or *db/db*) [10] display severe hyperphagia, obesity and disturbed glucose homeostasis.

In addition to the central actions of leptin, roles for the hormone have also been suggested in the periphery. Thus, LepR expression has been described in the endocrine pancreas [11,12], and several studies [12–18] have reported an action of leptin to inhibit insulin secretion. Furthermore, over-expression of insulin receptors in the islets of LepR-deficient Zucker diabetic fatty (ZDF) rats restores glucose-stimulated insulin secretion (GSIS) [19]. Whether physiological concentrations of leptin (≤1 nM) also inhibit secretion, however, has been contested [20].

Recent work [21] suggests that the actions of leptin on insulin release involve the stimulation of AMPK-activation protein kinase (AMPK) and trafficking of ATP-sensitive K^+^ (K_ATP_) channels to the plasma membrane. These changes are thus expected to impede the glucose-dependent closure of K_ATP_ channels, voltage-gated Ca^2+^ channel opening and Ca^2+^ influx that trigger insulin secretion in response to glucose [22].

Using a *Cre* deleter strain driven by the rat insulin 2 promoter (RIP2) Covey et al. [23] deleted critical *flox*'d exons in the gene encoding the active, long form of the leptin receptor (LepRb) *in vivo* in murine pancreatic β cells and in a specific population of neurons in the hypothalamus (RIP2*Cre* neurons). These animals developed obesity, fasting hyperinsulinaemia, impaired glucose-stimulated insulin release and glucose intolerance. On the other hand, Morioka et al. [24] disrupted the leptin receptor in all pancreatic cells using a *Pdx1* promoter-driven *Cre* and these mice, in contrast, exhibited normal body weight, improved glucose tolerance and elevated plasma insulin. However, when challenged with high fat diet, Pdx1*Cre*:LepR^F/F^ mice became glucose intolerant.

The difference between these studies is thus likely to be the result of using different *Cre* lines and hence deletion in partially overlapping, but different, cell types: crucially, in neither case was the leptin receptor deleted exclusively in pancreatic β cells. Thus, the RIP2*Cre* promoter used by Covey et al. is active in β cells and also in several areas of the brain, particularly in the hypothalamus [25]. By contrast, the Pdx1*Cre* used by Morioka et al. is expressed in adult β-cells and also deletes in glucagon-secreting pancreatic α cells as well intestinal glucagon-like peptide 1- (GLP-1) secreting L-cells and several other cell types with roles in glucose homeostasis [26]. The Pdx1*Cre* line also deletes in neuronal populations responsive to leptin [25].

Our first aim here, therefore, was to examine the effects of deleting the long form of the leptin receptor (LepRb) highly selectively in pancreatic β cells using the novel Ins1*Cre* deleter strain [27,28]. Selectivity with this strain is achieved firstly by the use of the Insulin *I* promoter, whose expression is essentially confined to pancreatic β cells [29]. By contrast, the Insulin *II* gene, which drives RIP2*Cre* promoter expression, is expressed in multiple brain regions [25,30]. Secondly, precise developmental and spatial control of *Cre* expression by Ins1*Cre* is enhanced by its targeted insertion (knock-in) at the *Ins1* locus. Finally, this line also avoids risks associated with the use of other insulin promoter-driven *Cre*s [31] of *Cre-*independent actions due to co-expression of growth hormone. The present strategy is expected, therefore, to distinguish between the actions of leptin on glucose homeostasis *via* the pancreatic β cell and those acting *via* the brain and elsewhere.

The possibility has also recently been explored that leptin receptors play a role in pancreatic α cells to control glucagon secretion [32]. However, whilst the latter studies failed to identify any defects in glucose homeostasis after LepR deletion using a *Cre* expressed under the control of a short fragment of the glucagon promoter [33], recombination was only achieved in ∼43% of α cells. Correspondingly, we [34] and others [35,36] have similarly found that the latter *Cre* deletes in only a minority (13–45%) of α cells. This leaves open the possibility that the effects of deletion described by Tuduri et al. [32] might be masked by changes in the remaining, non-recombined α cell population. To test this hypothesis, our second aim here was to use an alternative glucagon promoter-driven *Cre*, iGlu*Cre* [37], based on a much longer (∼100 kB) region upstream of the proglucagon gene, which drives recombination in the vast majority of α cells. However, *Cre* is also expressed with this line at other sites of proglucagon expression including the nucleus tractus solitarius (NTS) of the hindbrain [38], and in intestinal L-cells, both of which express leptin receptors [37,39]. Both of these cell types secrete the incretin glucagon-like peptide-1 (GLP-1) and thus impaired leptin receptor signalling, particularly in the neuronal population [40], might affect body weight, glucose homeostasis, or both.

## 2 Materials and methods

### 2.1 Generation of mice

Mice bearing LepR^F^ alleles on an FVB background were kindly provided by Dr Streamson Chua (Columbia University) and, after backcrossing twice to C57BL/6 mice, bred to Ins1*Cre* [28] or iGlu*Cre* [37] mice (C57BL6 background) as indicated. Further crosses to Rosa26tdRFP were performed with iGlu*Cre*LepR^F/F^ mice to allow labelling of recombined cells. Animals (2–5) were maintained in separately ventilated cages in a specific pathogen-free environment. All procedures were approved by the U.K. Home Office and were compliant with the Animals (Scientific Procedures) Act 1986 of the United Kingdom (PPL 70/7349). Genotyping was performed as described ([41] and see Results). Primer sequences are given in Table 1.

### 2.2 Studies of glucose homeostasis and hormone release *in vivo*

Intraperitoneal glucose (1 g/kg) or insulin (0.75 U/mL) tolerance tests were performed on mice fasted for 16 h or 5 h, respectively, as described [42], using an automated glucometer (Accucheck, Roche, Burgess Hill, U.K.). Plasma insulin and glucagon levels were measured with ELISA kits from Cis Bio (Bagnols-sur-Cèze, France) and Mercodia (Uppsala, Sweden), respectively.

### 2.3 Assay of insulin and glucagon secretion from isolated islets

Islet isolation by collagenase injection and pancreatic distension was performed as described [43]. Islets were cultured for 24–36 h in RPMI medium supplemented with 10% foetal calf serum, 100 U/mL penicillin and 100 μg/mL streptomycin. Hormone release was measured during 30 or 60 min (insulin or glucagon, respectively) static incubation of batches of six (insulin) or 20 (glucagon) islets in 0.5 mL modified Kreb's Ringer medium comprising (mM): 120 NaCl, 4.8 KCl, 24 NaHCO_3_, 0.5 Na_2_HPO_4_, 5 HEPES, 2.5 CaCl_2_, 1.2 MgCl_2_ and 5 d-glucose, pH 7.4, at 37 °C. Secreted and total hormone levels were measured by homogeneous time-resolved fluorescence-based (HTRF) assay (Cisbio).

### 2.4 Intracellular free Ca^2+^ imaging and connectivity analysis

Imaging was performed at 34–36 °C essentially as described [44]. Briefly, islets were loaded with Fluo2 (Teflab) and Ca^2+^ signals captured with a Nipkow spinning disk coupled to a Zeiss Axiovert M200 microscope fitted with x10–x20/0.3–0.5 numerical aperture (NA) objectives (EC Plan-Neofluar, Zeiss). Cells were illuminated at 491 nm using a solid state laser (Cobalt) with emitted light detected from 500 to 550 nm using a 16-bit back-illuminated EM-CCD (Hamamatsu C9100-13). Connectivity analysis was performed as described [44].

### 2.5 Immunocytochemical analysis

#### 2.5.1 tdRFP and glucagon

Animals were anaesthetised with ketamine and transcardially perfused-fixed with 4% PFA before post-fixation for 1 h and embedding in paraffin and sectioning. Sections were labelled with anti-RFP (Rockland, 600-401-379, 1:50 dilution, secondary-Alexa 568 1:250) and anti-glucagon (1:500 dilution, secondary-Alexa 488 1:250) and anti-glucagon (1:500 dilution, secondary-Alexa 488 1:250) antibodies and sealed using Vectashield hardset mounting medium with DAPI (Vector Laboratories).

#### 2.5.2 Insulin and glucagon

Mouse pancreata were extracted and fixed in 10% (v/v) neutral buffered formalin at 4 °C for 18 h before paraffin embedding and sectioning. Sections were labelled with polyclonal anti-insulin (Dako A4056, 1:200 dilution, Secondary-Alexa 488 1:1,000) or anti-glucagon (Sigma, G2654 or Santa Cruz, sc-13091, 1:200 dilution, Secondary-Alexa 568 1:500) antibodies and sealed using Vectashield hardset mounting medium with DAPI (Vector Laboratories). Fluorophores were excited using a Zeiss Observer Vivatome and a 20x/NA 0.8 x objective and 365 nm, 470-nm and 540–580-nm LEDs. Emission was detected using Semrock filters centred on 377/50 nm, 472/30 nm, and 534/20 nm for DAPI, Alexa 488, and Alexa 568, respectively.

### 2.6 Islet isolation and FACS sorting

Transgenic mice (males and females, >16 weeks) expressing the fluorescent proteins Venus under the control of the proglucagon promoter [45], or EYFP under the control of the prosomatostatin [46] promoter (>8 week old mice) were euthanized by cervical dislocation and the pancreas injected immediately with 5 ml of collagenase V (0.5 mg/ml) in Ca^2+^ and Mg^2+^-free HBSS. Pancreata were digested and the islets hand-picked into HBSS containing 0.1% fatty acid-free bovine serum albumin (BSA) and 11 mM glucose. Islets were disrupted into single cells by trituration following incubation for 1 min in Ca^2+^-free HBSS containing 0.1x trypsin/EDTA and 0.1% BSA. Cells were immediately sorted by flow cytometry using a BD Influx cell sorter (BD Biosciences, San Jose, CA, USA) equipped with a 488 laser for excitation of Venus or EYFP. To collect alpha and beta cells, Venus-positive and negative cells were collected, with the latter further subdivided into a population that was large (according to side and forward scatter) and with high background autofluorescence at 530 and 580 nm. To collect delta cells, only EYFP positive cells were collected. Cells were collected into RLT lysis buffer (QIAGEN) and frozen on dry ice.

### 2.7 Massive parallel sequencing (RNASeq)

Details of RNASeq for whole islets, and subsequent data analysis, are provided in [27]. For studies on individual islet cell types, total RNA was extracted using an RNeasy Micro Plus kit (Qiagen, Manchester, UK) according to the manufacturer's instructions. RNA was amplified using Ovation RNA-seq System V2 (PN 7102, NuGEN) whereby 2.5 ng of RNA for each sample was used (3, 2, and 4 replicates were used each for α, β, and δ cells, respectively, totalling 9 samples). To prepare the RNA-seq library, the amplified cDNA (3 μg per sample) was fragmented to 200 bp using the Bioruptor Sonicator (Diagenode) and barcode ligation and end repair were achieved using the Ovation Rapid DR Library System (PN 0319,0320, NuGEN). The barcoded libraries were combined and sent for SE50 sequencing using an Illumina HiSeq 2500 system at the Genomics Core Facility, Cambridge Institute, CRUK.

### 2.8 Quantitative RT-PCR

Total RNA was extracted using an RNeasy Micro kit (QIAGEN, UK), according to the manufacturer's protocol. Superscript III (Life technologies) reverse transcriptase was used to synthesize cDNA.

Analysis was performed with a 7900 HT Fast Real-Time PCR system (Applied Biosystems). The PCR reaction mix consisted of first-strand cDNA template, primer pairs for LepR, forward primer binding exon junction 16–17 (GTTTCACCAAAGATGCTATCGAC) and reverse primer binding exon 17 (GAGCAGTAGGACACAAGAGG), and PCR Master mix (Applied Biosystems). Expression of selected targets was compared with that of *Cyclophilin* measured on the same sample in parallel on the same plate.

### 2.9 Immunocytochemical analysis of Stat3 phosphorylation

Islets were incubated for 30 min in Kreb's Ringer bicarbonate buffer followed by incubation with either leptin (10 nM), IL1-β (50 ng/mL) and TNF-α (1000 pg/mL) or control conditions at 37 °C. Islets were fixed in 4% PFA for 1 h at 4 °C.

Antigen retrieval steps were performed by incubating islets in 0.01 M Tris-HCl for 5 min at 90 °C, before cooling to room temperature and incubation in 100% methanol at −20 °C for 10 min. Free-floating islets were stained immunohistochemically with anti-pSTAT3 (pTyr-705; NEB, 4113S, 1:100 dilution, secondary-Alexa 488 1:500) and anti-insulin (1:200 dilution, secondary-Alexa 488 1:500) antibodies, and mounted onto glass slides, as above. Images were obtained by using a Zeiss LSM-780 microscope and a 60x/NA 0.8 x objective.

### 2.10 Statistical analysis

Statistical significance was assessed using Student's t-test with Bonferroni correction, or two-way ANOVA, as indicated, using GraphPad Prism 6.0. P-values < 0.05 were considered significant. Values are presented as means ± S.E.M.

## 3 Results

### 3.1 LepR mRNA levels are low in isolated mouse islets and in purified islet cells

**Figure 1:**
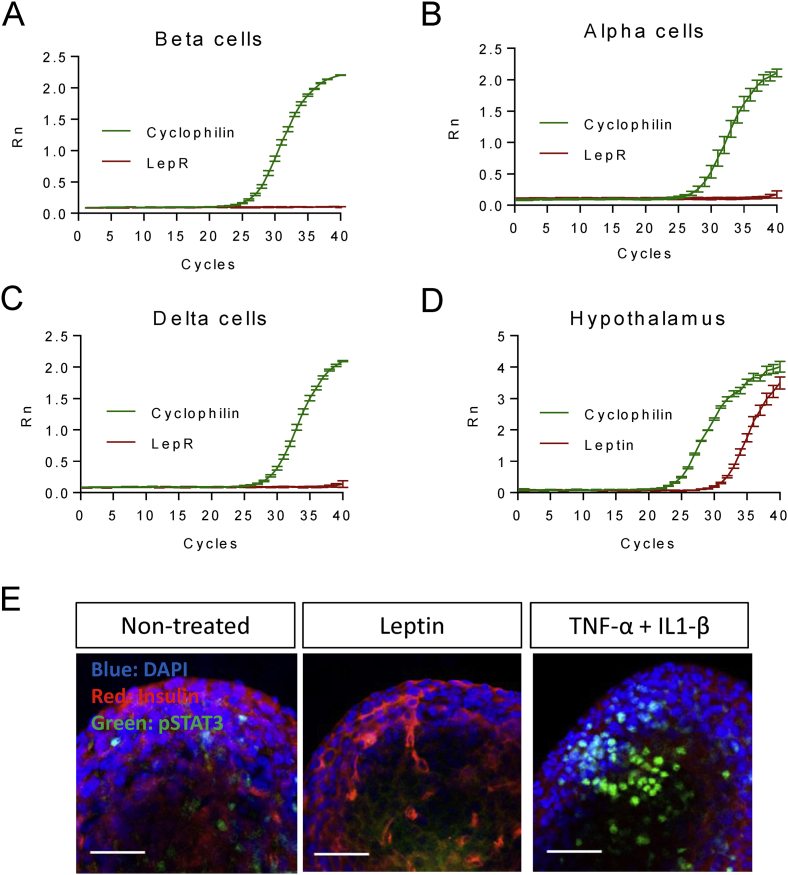
**Low levels of LepR mRNA and receptor signalling in purified islet cells and whole islets, respectively**. Amplification plot of *LepR* (red) or *Ppia* (cyclophilin, green) using mRNA purified from mouse islet **(A)** β **(B)** α or **(C)** δ cells or **(D)** hypothalamus. **(E)** Immunofluorescence staining of pStat3 in whole islets after treatment with either leptin (10 nM), TNF-α and IL1-β, or control non-treated islets. Data are representative of two further experiments. Scale bar 52.5 μm. See the Materials and Methods section for further details.

We first assessed expression of leptin receptors in isolated islets and highly purified preparations of islet cells from wild-type mice (Materials and Methods) using RNASeq and quantitative RT-PCR. LepR mRNA was detected at 0.2 RPKM in whole islets [27], at the lower 39th centile of all messages, and 0.23, 0.036 and 0 RPKM in purified β, δ and α and cells, respectively (*n* = 3, 2 and 4 separate preparations, respectively). Correspondingly, whereas qRT-PCR amplified *LepR* mRNA from whole islets (not shown), this approach failed to amplify a product from any of the three isolated cell preparations (Figure 1A–C). By contrast, the primers used efficiently amplified LepR mRNA from hypothalamus-derived polyA + mRNA (Figure 1D). Consistent with low levels of LepR protein in islets, staining of islets with fluorescently-labelled Cy3-leptin [12] failed to reveal consistent signals above background (results not shown). To further test the hypothesis that LepR signalling is low or undetectable in pancreatic islet cells, we exposed isolated islets from wild type C57BL6 mice to either leptin, or to a cytokine mixture (IL1-β and TNF-α) as a positive control, and monitored Stat3 phosphorylation on Tyr 705 using immunocytochemistry and laser-scanning confocal imaging. Whereas the cytokine mixture caused a robust increase in Stat3 phosphorylation as anticipated, no effects were observed upon addition of leptin, even at the highest dose (10 nM) tested (Figure 1E).

The above findings suggest that leptin receptor levels are vanishingly low (β, δ) or zero (α) in key islet cell types and limit the actions of the hormone on this tissue.

### 3.2 Gender and age-dependent effects of LepR deletion from pancreatic β cells

**Figure 2:**
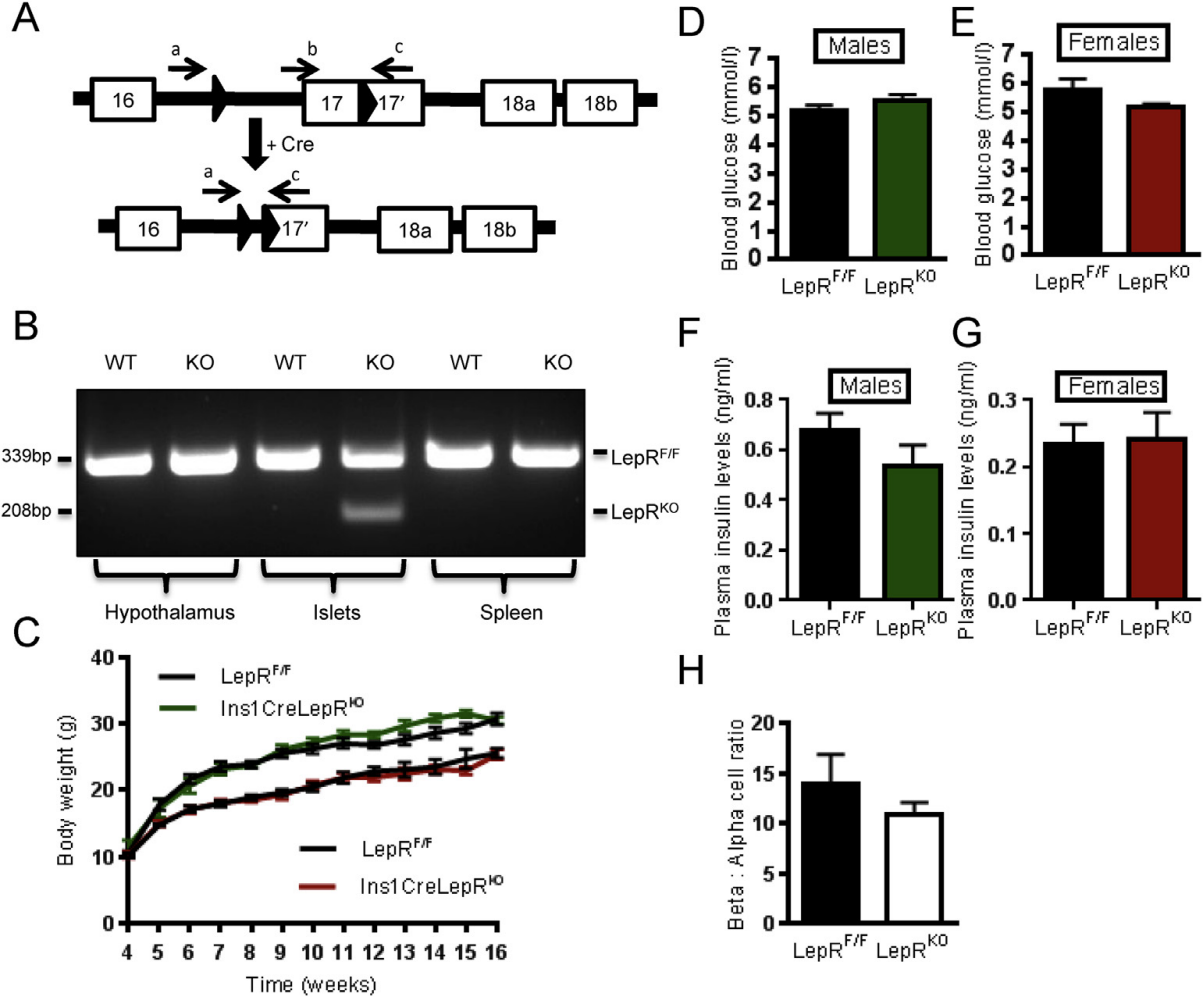
**Ins1*Cre* expression results in excision of the *flox*'d region of LepRb alleles selectively in islets**. Genomic DNA was harvested from Ins1*Cre*LepR^KO^ and control LepR^F/F^ animals and used as a template for PCR with the primers indicated **(A)**. Predicted product sizes are 339 bp for the *flox*'d allele (primers b,c) and 207 bp for the excised allele (primers a,c) **(B)**. Ins1*Cre*LepR^KO^ animals displayed a normal progression in bodyweight. Ins1*Cre*LepR^KO^ males (green line) and LepR^F/F^ (black line) and below Ins1*Cre*LepR^KO^ females (red line) and LepR^F/F^ (black line). **(C)**. Blood glucose concentration after overnight fast in males **(D)** (Ins1*Cre*LepR^KO^, *n* = 8, LepR^F/F^, *n* = 7) and females at 8 weeks **(E)** (Ins1*Cre*LepR^KO^, *n* = 13, LepR^F/F^, *n* = 9). Plasma insulin levels in male mice **(F)** Ins1*Cre*LepR^KO^, n = 10, LepR^F/F^ n = 7, and in females **(G)** Ins1*Cre*LepR^KO^, *n* = 9, LepR^F/F^, *n* = 7. β to α cell ratio in Ins1*Cre*LepR^KO^ and wild-type mice **(H)**.

To ablate residual LepRb function selectively from pancreatic β cells, animals bearing alleles in which *Lox*P sites flank exon 17 of the leptin receptor gene [41] were crossed with animals harbouring the Ins1*Cre* transgene. Exon 17 includes the BOX1 domain required for leptin-induced JAK-STAT signalling [41]. Based on the use of reporter strains [27], this approach is expected to lead to recombination in the majority (>94%) of β cells, with <2% recombination in other islet cell types [27], in the resulting Ins1*Cre*LepR^KO^ mice. To confirm *Cre*-mediated excision of exon 17 (LepRb^Δ17^), genomic DNA was isolated from several tissues and PCR performed with two sets of primers as indicated in Figure 2A [41]. PCR amplification of DNA from islets derived from LepRb^F/F^ or Ins1*Cre*LepR^KO^ animals generated a product of 339 bp and a band of 208 bp where *Cre*-mediated excision of exon 17 had occurred (Figure 2B). Whilst attempts were made to confirm deletion by measuring full-length and truncated LepRb mRNA, levels these were below the level of reliable quantitation both in LepRb^F/F^ and Ins1*Cre*LepR^KO^ mice, as discussed above, and in line with the absence of a functional effect of leptin on islet pStat3 phosphorylation (Figure 1E).

Consistent with the absence of recombination and deletion of LepRb in satiety centres in the hypothalamus, loss of LepRb mediated by Ins1*Cre* in the β cell exerted no effect on body mass in Ins1*Cre*LepR^KO^ mice (Figure 2C). Neither fasting glucose (Figure 2D,E) nor plasma insulin (Figure 2F,G) was different between wild-type and null mice. Since both the previously-described leptin receptor-deficient mice generated by recombination with RIP2*Cre* [23] or Pdx1*Cre* [24] animals display an abnormal β cell area, β to α cell ratio was examined in Ins1*Cre*LepR^KO^ animals. No changes in this parameter were apparent (Figure 2H).

**Figure 3:**
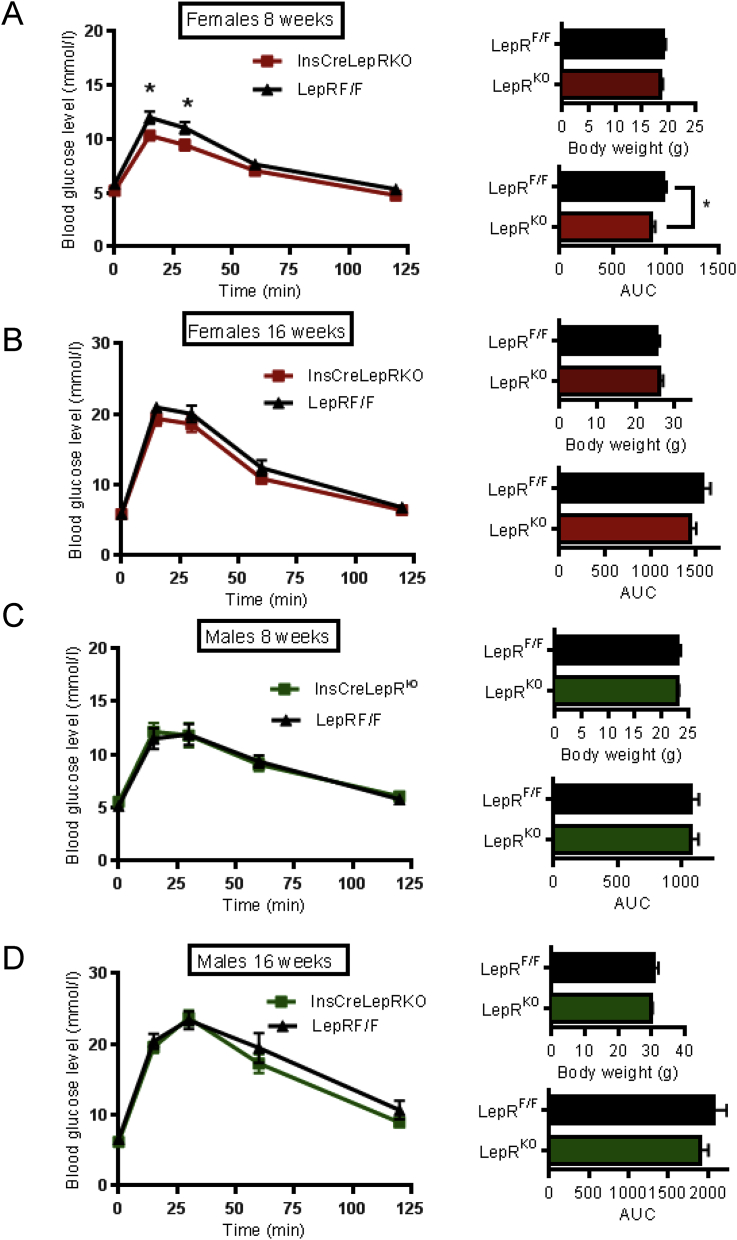
**Ins1*Cre*LepR**^**KO**^**females display improved glucose tolerance at the age of 8 weeks**. **(A)** Blood glucose concentration following IPGTT in Ins1*Cre*LepR^KO^ (square symbol) and LepR^F/F^ littermate controls (triangles) in 8-week old females: Ins1*Cre*LepR^KO^, *n* = 13, LepR^F/F^, *n* = 9, **(B)** 16-week old females: Ins1*Cre*LepR^KO^, *n* = 15, LepR^F/F^, *n* = 17, **(C)** 8-weeks old males: Ins1*Cre*LepR^KO^, *n* = 8, LepR^F/F^, *n* = 7, **(D)** 16-week old males: Ins1*Cre*LepR^KO^, *n* = 13, LepR^F/F^, *n* = 10. For all panels, the corresponding body weight and area under curve (AUC) are inset. Data are expressed as the average ± SEM. Statistical analysis was performed using two-way ANOVA *P < 0.05.

**Figure 4:**
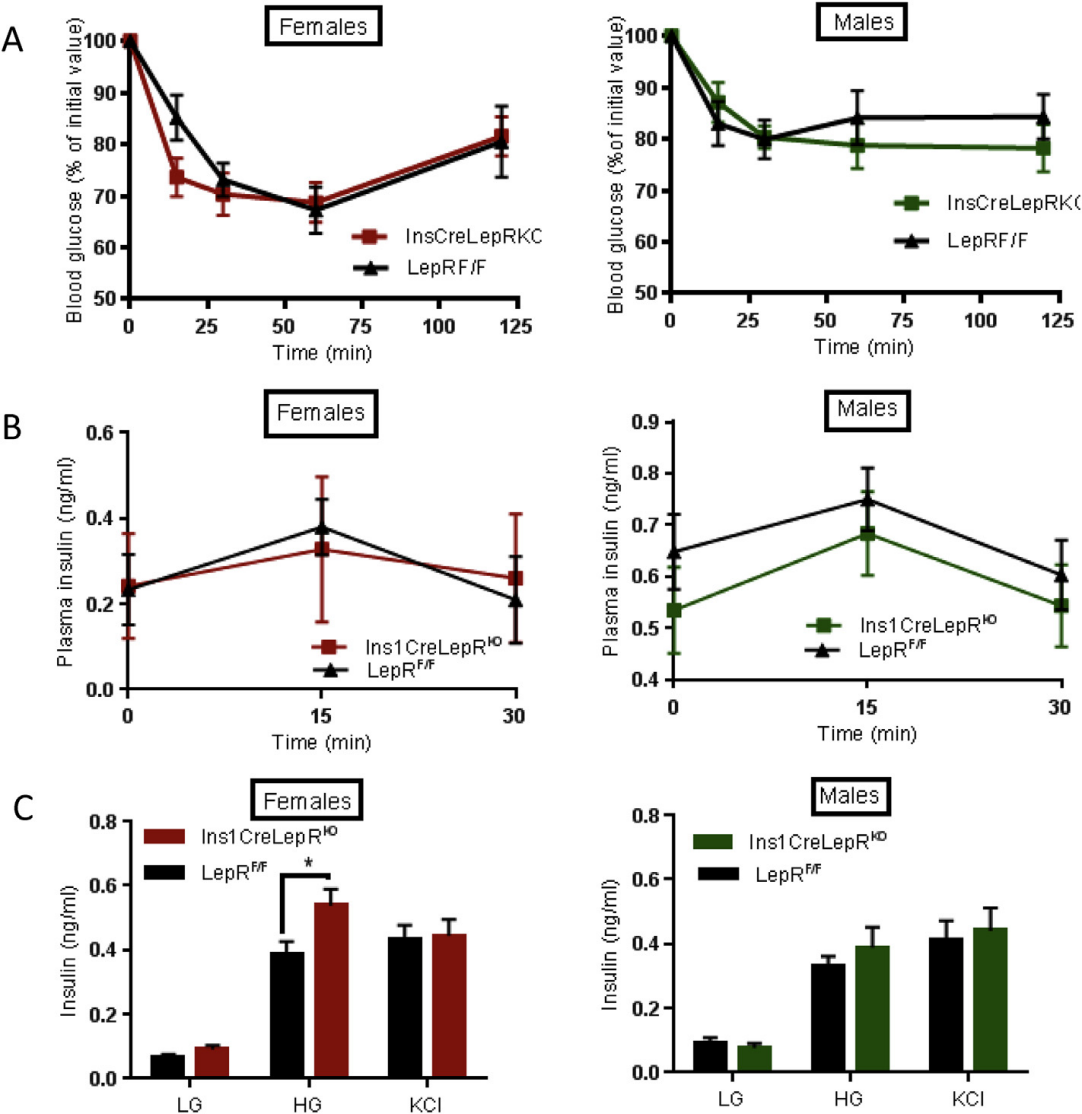
**Enhanced glucose-stimulated insulin secretion in Ins1*Cre*LepR**^**KO**^**females. (A)** Blood glucose after IP injection of insulin in Ins1*Cre*LepR^KO^ (square symbol) and LepR^F/F^ littermate controls (triangles) in males and females (*n* = 9–10 mice per genotype). **(B)** Plasma insulin levels after IP injection of glucose in males and females: Ins1*Cre*LepR^KO^, *n* = 10, LepR^F/F^, *n* = 6. **(C)** Insulin secretion as assessed from isolated islet in response to glucose (LG = 3 mM, HG = 17 mM) and KCl (30 mM) in 30-week old female and male mice. *n* = 3 separate experiments involving 2–3 mice per genotype/experiment. Statistical analysis was performed using two-way ANOVA *P < 0.05.

To examine in more detail whether disruption of leptin signalling in β cells affected glucose metabolism, animals aged 8, 12 and 16 weeks were subjected to intraperitoneal glucose tolerance tests (IPGTT). At 8 weeks, female Ins1*Cre*LepR^KO^ null mice showed transiently improved glucose tolerance, which reversed by 16 weeks (Figure 3A,B). No glycaemic phenotype was identified in male Ins1*Cre*LepR^KO^ at any of the ages examined (Figure 3C,D). Ins1*Cre*LepR^KO^ mice also displayed normal insulin sensitivity (Figure 4A).

### 3.3 Impact of LepRb deletion on β cell glucose signalling and insulin secretion

Given the reported role of leptin to inhibit insulin secretion [23,24], we measured insulin secretion in Ins1*Cre*LepR^KO^ mice *in vivo* and *in vitro*. No differences in the excursions in plasma insulin concentration were observed during intraperitoneal glucose tolerance tests between Ins1*Cre*LepR^KO^ mice and littermate controls (Figure 4B). Glucose- (16.7 vs 3 mM) stimulated insulin secretion was significantly potentiated *in vitro* in LepRb-deleted islets from 30 week old female, but not male Ins1*Cre*LepR^KO^ mice (Figure 4C). These changes, which may reflect the lower variability of measurements performed in isolated islets, are consistent with the observed small improvements in glucose tolerance (Figure 3A).

**Figure 5:**
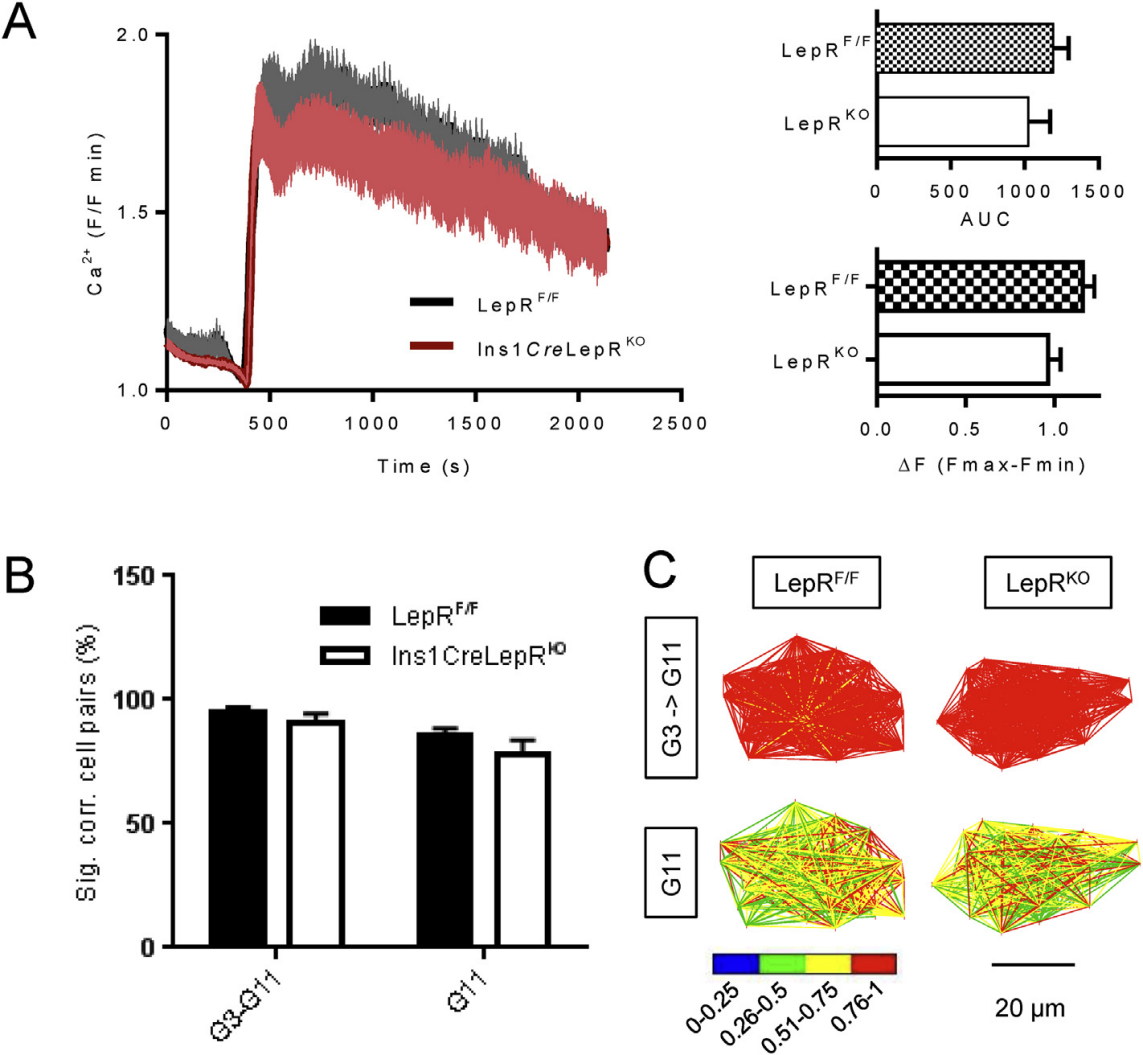
**Ins1*Cre*LepR**^**KO**^**islets display impaired Ca**^**2+**^**responses to high glucose**. Ca^2+^ recordings from control LepR^F/F^ islets (black line) and Ins1*Cre*LepR^KO^ islets (red line) in response to either 11 mM glucose **(A)**. Inset are area under the curve (AUC) and amplitudes (ΔF (F_max_-F_min_)) of the Ca^2+^ rises. Data are from 14 to 16 islets (3–4 mice) per genotype. **(B,C)** Connectivity analysis [44] showing the proportion (%) of significantly correlated cell pairs during a step change from 3 to 11 mM glucose (*i.e*. the activity onsets), or at steady state in the continued presence of 11 mM glucose. Pseudocolor plots in **(C)** show the strength of connections, determined using by Pearson Correlation (Pearson R). Data are expressed as mean ± SEM. Statistical analysis was performed using two-way ANOVA and Student's t-test. *P < 0.05.

Deletion of LepRb from β-cells using a RIP2*Cre* has been reported to impair glucose-dependent Ca^2+^ signals [47]. However, this observation was ascribed partly to the effects of the RIP2*Cre* transgene itself [31], as well as to the loss of LepRb. Contrary to the latter findings, deletion of LepRb with Pdx1.*Cre* potentiated glucose-induced Ca^2+^ oscillations. To examine the effect of LepRb deletion more selectively in the β cell we therefore monitored changes in intracellular free Ca^2+^ ([Ca^2+^]_i_) in response to high glucose, or depolarisation with KCl, in islets from 8 weeks old female Ins1*Cre*LepR^KO^ or control mouse islets. Islets lacking LepRb in β cells displayed a tendency towards lower [Ca^2+^]_i_ increases in response to high glucose (Figure 5A), though this difference was not significant when hundreds of single cells were individually assessed within each micro-organ to exclude outliers which confound signal analysis (i.e. glucose non-responsive cells, and those cells displaying large and sustained increases indicative of apoptosis). Likewise, no changes in response to stimulation with KCl (not shown), or in β cell-β cell connectivity (Figure 5B,C), [44], were observed in Ins1*Cre*LepR^KO^ mice *versus* control littermates suggesting that β cell population responses to glucose remain coordinated even in the absence of LepRb signalling.

### 3.4 Role of leptin receptors in pancreatic α- and other proglucagon-expressing cells

Leptin has been reported to exert a direct inhibitory effect on glucagon secretion from isolated mouse and human islets [48]. To generate animals deleted for the LepRb in pancreatic α cells and other proglucagon-expressing cells (including GLP-1-expressing intestinal L-cells and GLP-1^-^positive neurons in the brain), LepRb *flox*'d animals [41] were crossed with animals harbouring the iCre recombinase transgene under the control of ∼100 kB region immediately upstream of the proglucagon gene [37]. The resulting iGlu*Cre*LepR^KO^ mice were further crossed to Rosa26.tdRFP reporter mice [49].

**Figure 6:**
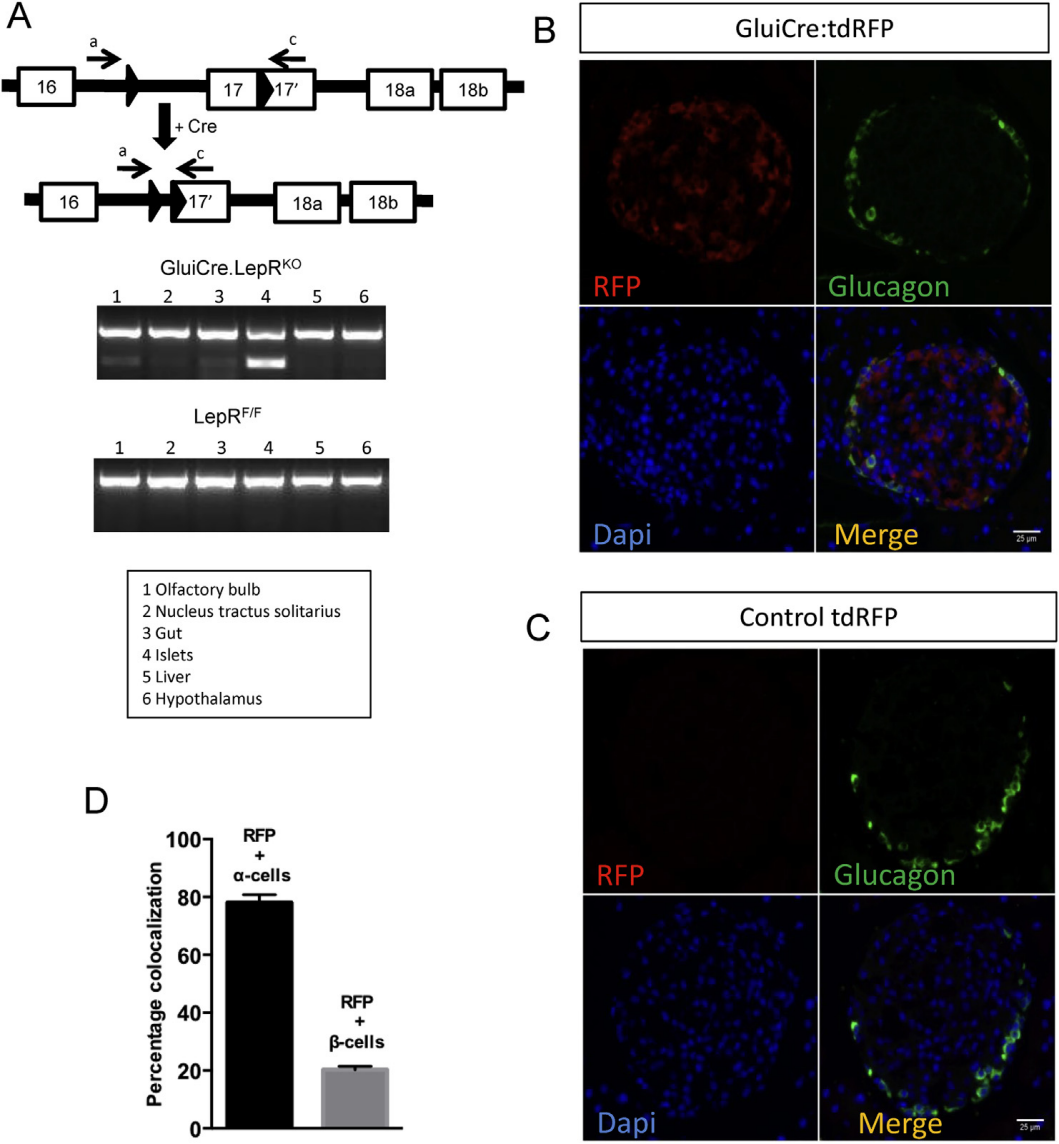
**iGlu*Cre* expression results in recombination of LepRb *flox*'d alleles in the olfactory bulb, nucleus tractus solitarius, intestine and pancreatic islets**. Genomic DNA was harvested from the tissues indicated and used as a template for PCR with primer pair a and c (as indicated in Figure 1A). Predicted product sizes are 625 bp for the *flox*'d allele and 207 bp for the excised allele. **(A)** PCR transcript of the excised alleles in iGlu*Cre*LepR^KO^ and LepR^F/F^ controls. Immunohistochemical analysis of pancreas from iGlu*Cre*:tdRFP^StopFlox^ mice, bearing wild type LepR alleles **(B)** stained for RFP (red), glucagon (green) and DAPI (blue), and in control animals without *Cre* (tdRFP^StopFlox^) **(C)**. Quantification of the percentage of RFP expressing α and β cells **(D)**. *n* = 39 islets from three animals. Data are expressed as mean ± SEM.

In order to confirm firstly the deletion of LepRb in target cells, genomic DNA was harvested from several different tissues and brain areas and PCR was performed using primers flanking the LoxP sites (Methods; Figure 6A). The presence of the recombined allele was clearly detected in islets, and to a lesser extent in the olfactory bulb, the nucleus tractus solitarius (NTS) and intestine. Correspondingly, in mice carrying the Rosa26.tdRFP reporter, red fluorescence was clearly apparent in the majority of glucagon-positive α cells (Figure 6B), but not in Rosa26.tdRFP mice lacking iGlu*Cre* (Figure 6C). In the presence of the *Cre* transgene, recombination occurred in the vast majority (∼80%) of glucagon-positive islet cells, but also in a minority (∼20%) of insulin-positive β cells (Figure 6B,D).

**Figure 7:**
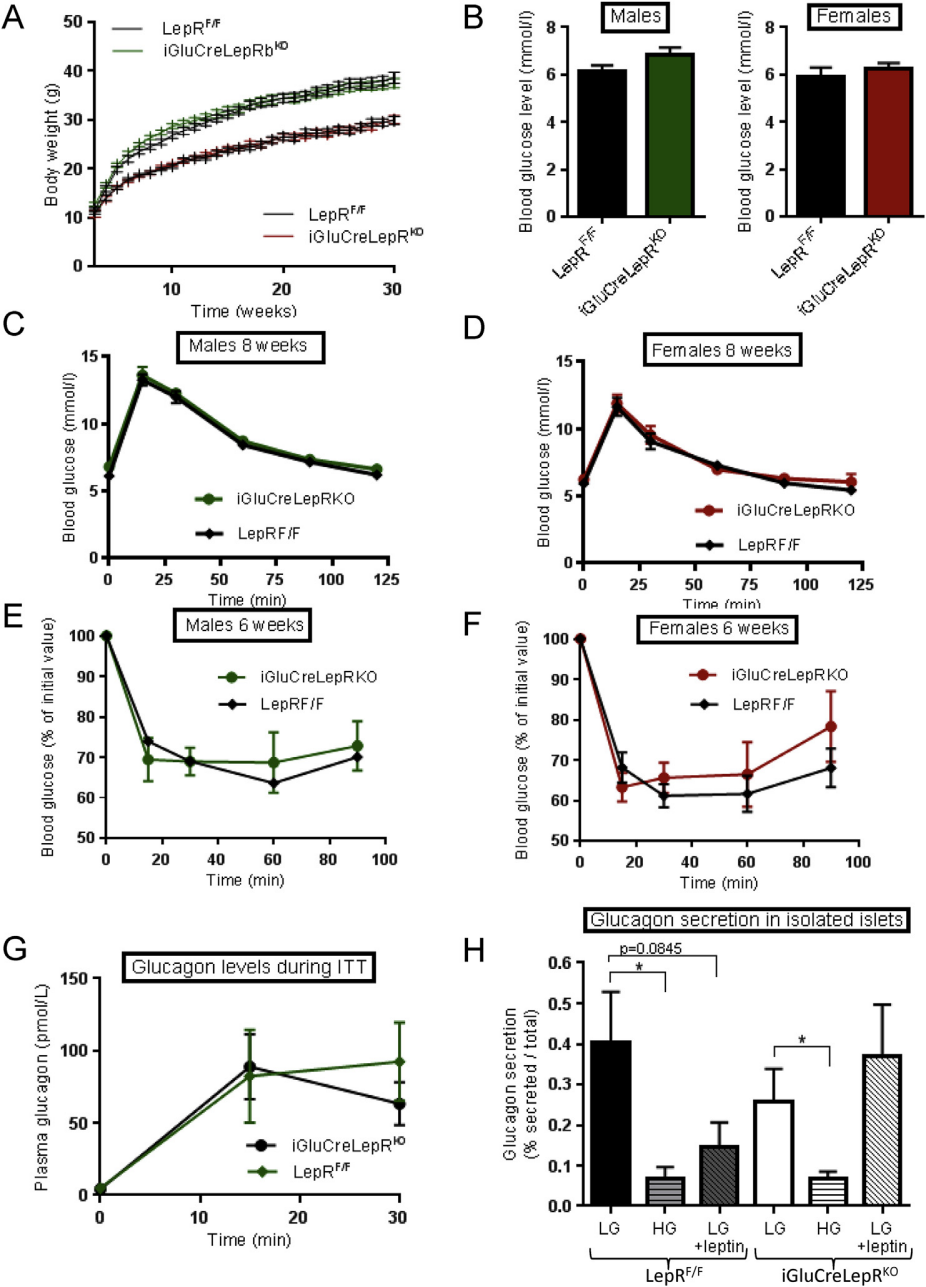
**iGlu*Cre*LepR**^**KO**^**mice display normal glucose tolerance and fasting blood glucose levels. (A)** Body weight followed for 30 weeks in male (green) and female (red) iGlu*Cre*LepR^KO^ and LepR^F/F^ mice (*n* = 12–19 mice per genotype). **(B)** Fasting blood glucose levels in 8 week old males and females (*n* = 6–11 mice per genotype). **(C,D)** Blood glucose concentration after IPGTT in male and female iGlu*Cre*LepR^KO^ (square symbol) and LepR^F/F^ (green triangle) mice. Blood glucose concentration after ITT in males: **(E)** iGlu*Cre*LepR^KO^, *n* = 8, LepR^F/F^, *n* = 7; and females: **(F)** iGlu*Cre*LepR^KO^, *n* = 8, LepR^F/F^, *n* = 10. Plasma glucagon levels during ITT, iGlu*Cre*LepR^KO^, *n* = 6, LepR^F/F^, *n* = 4, **(G)**, and glucagon release from isolated islets in the presence of the indicated glucose concentrations: LG = 0.5 mM, HG = 10 mM glucose; *n* = 6 animals per genotype **(H)**. When present, leptin was added to 10 nM. Data are expressed as the mean ± SEM and statistical comparison was through two-way ANOVA, *p < 0.05. Other details are provided in the Materials and Methods section.

No differences in growth curves were apparent between iGlu*Cre*LepRb^KO^ and LepRb^F/F^ mice (Figure 7A), nor were differences in fasting blood glucose detected (Figure 7B). Intraperitoneal glucose (Figure 7C,D) and insulin (Figure 7E,F) tolerance were also identical between genotypes. Likewise, there were no apparent differences in glucagon release in response to hypoglycaemia *in vivo* (Figure 7G), nor *in vitro* in response to low or high glucose (Figure 7H). A tendency for leptin (10 nM) to impair glucagon release at low glucose was apparent in wild-type, but not iGlu*Cre*LepRb^KO^ mice (Figure 7H).

## 4 Discussion

### 4.1 Leptin receptor signalling in β cells

The studies described herein present the first examination to our knowledge of the impact of highly selective abrogation of leptin receptor function in pancreatic β cells using Ins1*Cre*-mediated deletion. We show firstly that levels of leptin receptor mRNA and of leptin signalling are vanishingly low in highly purified mouse islet cells and whole islets, respectively (Figure 1). These results corroborate work from Fujikawa, Coppari and colleagues [50] who showed, through the use of a leptin receptor promoter *Cre* crossed to Rosa26RFP reporter mice, that RFP expression (reflecting the activity of the LepR promoter) is essentially undetectable in the endocrine pancreas, both during development and in adult mice. In contrast, recombination was detected in a more poorly-defined cell population outside of the islet, possibly corresponding to duct cells or ductal precursors. Importantly, the latter cells are likely to contaminate islets after isolation, and, as such, may reflect the major pool of LepRb-expressing cells in these *ex vivo* preparations. Consequently, the use of *Cre* deleter strains active throughout the pancreas, notably Pdx1Cre, is expected to cause recombination and loss of LepRb from these contaminating (non-islet) cells. The loss of LepRb mRNA apparent in islets from the above mice in a previous report [24], therefore, seems likely to reflect deletion from the above population other than from β-cells *per se*. We note that the report by Covey et al. [23] using the Rip2*Cre* deleter strain, and therefore expected in the pancreas to delete only in β cells, does not include any data on LepRb mRNA levels in wild type *versus* null mouse islets. Only measures of DNA recombination, as performed in our own study, were provided.

Nevertheless, and consistent with earlier findings using more promiscuous *Cre*s [23,24], we show that the levels of leptin receptors on β cells may be sufficient to restrict β cell function *in vivo* under some circumstances. Thus, we report mild glycaemic effects of LepRb deletion which appeared transiently in female mice (Figure 3A,B). Although challenged by the findings of Coppari and colleagues [50] (above) one possibility, not explored here, is that this might reflect a transiently higher level of expression of leptin receptors on β cells at this developmental stage in females. However, the alternative possibility that a very low level of LepR expression, perhaps on a minority of well-connected (“pacemaker” or “hub”) β cells [51], is sufficient to exert islet-wide consequences on insulin secretion in juvenile females, cannot be discounted.

We note that, whilst in one previous report [23] the effects of β cell-targeted LepRb deletion were examined using oral glucose tolerance tests (OGTTs), in a second paper [24], equally dramatic effects of deleting these receptors throughout the pancreas were observed using IPGTTs, where peak blood glucose was reduced from ∼22 to 16 (27%) and from >16 to ∼9.5 mM (40%) in 6 month old males and females, respectively. Our own experiments, using the more selective Ins1Cre driver line to delete LepRb, revealed a much more modest reduction in peak glucose during IPGTT, from ∼12 to 10 mM (16%) in 8 week old females, whereas no differences were observed between control and knockout male mice of the same age, or between 4 month old mice of either sex (Figure 3). The measurements in the present study are thus best compared to the latter report [24], using IPGTTs, and support our contention of a limited role for LepR in β cells beyond juvenile stages. The more marked effects of LepRb deletion described in earlier studies [23,24] would seem to be attributable, at least in large part, to the loss of leptin receptors at other sites, notably the brain, with consequent effects on food intake (and hence insulin sensitivity) [23] or potentially in other non-endocrine pancreatic cells [24]. Interestingly, Morioka et al. [24] did not observe changes in body weight after Pdx1*Cre*-mediated deletion, whilst markedly elevated insulin secretion and β cell mass were reported.

A potential concern with the present studies is that deletion may not have occurred throughout the β cell complement, as we have assumed [27]. Whilst *LepRb* mRNA levels were too low to quantitate accurately either in wild type or Ins1*Cre*LepR^KO^ islets (Figure 1A–C), we believe this possibility to be unlikely. Firstly, we observed clear recombination at the genomic level in isolated islets (Figure 2B). Secondly, Ins1*Cre* leads to highly efficient recombination at *Lox*P sites in other targets including in LKB1/STK11, AMPKα1 and α2 alleles [27], at the Rosa26-eYFP locus [28], and in mice carrying a single halorhodopsin allele downstream of a *Lox*P-STOP-*Lox*P cassette (D.J.H. and G.A.R. results not shown). In the two latter models, recombination is observed in 97.8% and 94.7% of β cells, respectively.

Further supporting a limited role of LepRb on β cells are studies by Chua and colleagues [52] demonstrating an absence of metabolic phenotype in mice deleted selectively for LepR in the periphery. In light of the above observations, our own data in Ins1*Cre*LepR^KO^ animals reporting only a weak metabolic phenotype are not entirely unexpected.

Interestingly, the inhibitory actions of leptin observed in the present study appear to be independent of glucose-induced increases in intracellular Ca^2+^, since the latter showed only a non-significant tendency to be *impaired* in Ins1*Cre*LepR^KO^ mice (Figure 5).

### 4.2 Leptin receptor signalling in α cells

Using the less efficient PPG.*Cre* [33], earlier studies [32] have reported that elimination of LepRb from a subset of α cells exerted no effect on glucose tolerance. However, and consistent with findings from Tuduri et al. [48], we observed a tendency for leptin to inhibit glucagon secretion *in vitro* (Figure 7G), an effect which was abolished in iGlu*Cre*LepRb^KO^ mouse islets. Similarly, a tendency was observed towards a more rapid recovery towards normal glycaemia after insulin injection in female KO mice (Figure 7F). Thus, at least under the conditions examined here, leptin receptor signalling in α cells has no more than a marginal role at most in the control of glucagon secretion during hypoglycaemia.

### 4.3 Leptin receptors in GLP-1 neurons

In the present study, we failed to observe differences in weight gain between wild-type and iGlu*Cre*LepRb^KO^ mice. Whilst the simplest interpretation of these data would be that GLP-1-expressing neurones in the NTS are not involved in the control of food intake, we note that Scott and colleagues [53] recently observed that disruption of LepRb signalling in this brain region using a less selective Phox2B deleter strain led to hyperphagia but not weight gain. This was attributed to an increase in energy expenditure in null mice. Whether deletion of LepRb using the iGlu*Cre* transgene similarly affected food intake is a possibility which will require testing in individually-housed mice in the future.

### 4.4 Leptin receptors in other proglucagon-expressing cells

Intestinal L-cells, which synthesise and secrete GLP-1, are also an important site of proglucagon expression, and consequently recombination in iGlu*Cre* mice [37,38]. Although not measured directly in the present studies, changes in GLP-1 output from these cells would seem to be unlikely in iGlu*Cre*LepR^KO^ mice given their unaltered body weight gain (Figure 7A) and glucose tolerance (Figure 7C,D), as well as the low expression of leptin receptor mRNA in FACS-purified mouse L-cells (FR and FMG, data not shown).

## 5 Conclusions

Using a well-defined and efficient *Cre* deleter strain we demonstrate that leptin signalling in β cells plays a physiologically relevant, albeit very limited role, to control insulin secretion *in vivo*. Similarly, leptin has a relatively minor, if any, role in the control of glucagon secretion from pancreatic α cells. No direct evidence was obtained in the present study for a role for leptin receptors in GLP-1-expressing cells in the gut or brain.

## Figures and Tables

**Table 1:** Primer sequences.

Target	Forward primer sequence 5′-3′	Reverse primer sequence 5′-3′
Tissue control	FER2-Q2: accttcagaccttggcgttggagg	FER2-R2: cctgaggttcctgttgctgtgactcc
Ins1Cre	Cre jva rv 2: gccagattacgtatatcctggcag	Cre jva fw 2: ttcactggttatgcggcgg
LepR^F^	103: tgagttccctcatgattctgg	See below
LepR^F^	104: cagccgaccaatgcttatt	105: acaggcttgagaacatgaacac
iCre	Cre002: gacaggcaggccttctctgaa	Cre003: cttctccacaccagctgtgga
β-catenin	RM41: aaggtagagtgatgaaagttgtt	RM42: caccatgtcctctgtctattc
tdRFP	Anti: ctacaggaacaggtggtgg	Sense: ctgttcctggggcatggc
